# Effects of Birthing Room Design on Maternal and Neonate Outcomes: A Systematic Review

**DOI:** 10.1177/1937586720903689

**Published:** 2020-02-20

**Authors:** Christina Nilsson, Helle Wijk, Lina Höglund, Helen Sjöblom, Eva Hessman, Marie Berg

**Affiliations:** 1 Faculty of Caring Science, Work Life and Social Welfare, University of Borås, Sweden; 2 Institute of Health and Care Sciences, Sahlgrenska Academy, University of Gothenburg, Sweden; 3 Quality Assurance and Patient Safety Unit, Sahlgrenska University Hospital, Gothenburg, Sweden; 4 Department of Architecture, Chalmers University of Technology, Gothenburg, Sweden; 5 Obstetric Unit, Sahlgrenska University Hospital, Gothenburg, Sweden; 6 Biomedical Library, University of Gothenburg, Sweden

**Keywords:** systematic literature review, childbirth, birthing room, healthcare environment, design, maternal and neonate outcomes, evidence-based design (EBD), outcomes—design, labor and delivery units

## Abstract

**Aim:**

To summarize, categorize, and describe published research on how birthing room design influences maternal and neonate physical and emotional outcomes.

**Background:**

The physical healthcare environment has significant effects on health and well-being. Research indicates that birthing environments can impact women during labor and birth. However, summaries of the effects of different environments around birth are scarce.

**Methods:**

We conducted a systematic review, searching 10 databases in 2016 and 2017 for published research from their inception dates, on how birthing room design influences maternal and neonate physical and emotional outcomes, using a protocol agreed a priori. The quality of selected studies was assessed, and data were extracted independently by pairs of authors and described in a narrative analysis.

**Results:**

In total, 3,373 records were identified and screened by title and abstract; 2,063 were excluded and the full text of 278 assessed for analysis. Another 241 were excluded, leaving 15 articles presenting qualitative and quantitative data from six different countries on four continents. The results of the analysis reveal four prominent physical themes in birthing rooms that positively influence on maternal and neonate physical and emotional outcomes: (1) means of distraction, comfort, and relaxation; (2) raising the birthing room temperature; (3) features of familiarity; and (4) diminishing a technocratic environment.

**Conclusions:**

The evidence on how birthing environments affect outcomes of labor and birth is incomplete. There is a crucial need for more research in this field.

To give birth is a central life event that women will remember and be affected by throughout their lives (Parratt, 2002; Simkin, 1991). It is an innate, biological, and instinctive process, but one which has always been linked to certain risks (Macuhova et al., 2002). Therefore, mammalian mothers have instinctively always chosen to give birth in an environment perceived as safe, secure, and private (Macuhova et al., 2002; Newton, 1992).

A birthing woman needs both high-quality care that minimize risks of complications and a familiar, calm, safe, and secure environment so her hormonal system can function optimally and facilitate a physiological and healthy labour and birth (Foureur, 2008; Macuhova et al., 2002). The birthing woman’s hormonal system can be strongly influenced by stress, and the stress levels are influenced by the environment. An environment perceived as safe will reduce stress and facilitate the release of endogenous oxytocin (Uvnäs-Moberg, 2014).

***A birthing woman needs both high-quality care that minimize risks of complications and a familiar, calm, safe, and secure environment so her hormonal system can function optimally and facilitate a physiological and healthy labour and birth***.

We know that the physical healthcare environment has significant effects on health and well-being. Physical aspects such as good ventilation, windows, conditions that promote orientation and distraction, a view of or access to nature, real or artificial, and ergonomic furniture can all have positive health effects (Ulrich, 2019; Ulrich et al., 2008). However, the impact and effect of the physical care environment in relation to labor and birth is insufficiently understood.

Today, most women give birth in hospitals with a mostly unfamiliar medically equipped environment, sometimes noisy, and brightly lit (Stenglin & Foureur, 2013). Such environments can upset the balance between sympathetic and parasympathetic activity, leading to an increased activity of the body’s stress system and a disturbed release of endogenous oxytocin. This can in turn negatively influence the ability of the woman’s uterus to contract effectively, slowing or even stopping the labor process, and having a negative impact on blood supply and thus oxygenation to the fetus (Macuhová et al., 2002; Uvnäs-Moberg, 2014). This in turn leads to medical intervention (Foureur, 2008), which, together with a negative childbirth experience, has been linked to fear of giving birth again (Dencker et al., 2019; Nilsson, 2014). The opposite also applies; if the woman perceives the environment as safe, harmless, friendly, and inviting, physical and mental relaxation ensues, and fear and stress responses decease. Labour contractions become more effective and uterine blood circulation improves. This positively affects the birth progress, increases oxygenation of the foetus, and prevents postpartum haemorrhage (Sato et al., 1996; Uvnäs-Moberg et al., 2005).

The effect of healthcare environment on labor and birth has been only sparingly studied. A survey of 2,000 new mothers in Britain indicated that the physical environment could have both positive and negative effects on childbirth. Aspects of the birthing room identified as important were as follows: a nonclinical impression, a nicely decorated space, soundproofing so that people outside could not hear, possibility to control who had access to the room, possibility to control the temperature and the light, comfortable pillows and seating, rugs on the floors, and a private toilet, bath, and shower (Newburn & Singh, 2003).

We have only found one scoping review on whether the physical birthing environment impacts interventions during labor and how this might occur (Setola et al., 2019). The relative lack of research to support a direct connection between the physical birthing environment, and the rate of medical interventions, led to a broadened search of how the physical characteristics in design may indirectly influence interventions during labor and birth. This resulted in a description of eight types of building spaces that require more research (Setola et al., 2019). These spaces covered the design and layout of the labor ward, including the midwives’ desk, social rooms, and aspects of the birthing rooms (size, filter, shape, sensory elements, etc.), as well as birth philosophy. The study by Setola et al. (2019) contributes to an understanding of how the physical birth environment can indirectly influence intervention rates through the behavior, experiences, and practices of women and health professionals.

The effects of different models of care during labor and birth have been scientifically explored (Sandall et al., 2016), demonstrating that the rate of medical interventions can also be related to the organization of maternity care. It is, for instance, more beneficial to give birth in a midwifery-led unit than on a standard hospital labor ward because of the lower occurrence of medical technical interventions, but also in terms of more spontaneous vaginal births, fewer instrumental births, and higher satisfaction in healthy women with normal pregnancies, all without any difference in rates of negative effects (Hollowell et al., 2017; Overgaard et al., 2012; Sandall et al., 2016).

To summarize, the physical healthcare environment has significant effects on patients’ health and well-being, but the influence of the physical environment on labor and birth has not been well-studied. There is only one review on how birthing environments may indirectly impact interventions during labor. The direct effects of physical birthing environments on health outcomes in women and neonates have not been systematically summarized. We wanted to explore the research field further, by doing a wide (including all outcomes) and deep (limited to birthing room design) search on the basis of the following research question: What does the literature say regarding the influence that birthing room design has on maternal and neonate physical and emotional outcomes? We therefore conducted a systematic review to summarize, categorize, and describe published research on how birthing room design influences maternal and neonate physical and emotional outcomes. This review will include all studies that focus on aspects of the birthing room that have been explored and related to labor and birth outcomes in either women giving birth or their babies. Thus, the result of this study will partly overlap the result by Setola et al. (2019), but also bring complementary findings that provide additional knowledge to guide the design of birthing rooms.

## Method

### Search Strategy and Selection of Papers

For this systematic review, the following criteria were stated for inclusion and exclusion of literature. Criteria for inclusion were all types of peer-reviewed scientific papers which examined all health outcomes and experiences in women and their babies during labor and birth as well as shortly after birth. In December 2016, we searched the following electronic bibliographic databases because of their relevance to the topic of research, from their inception dates: Avery Index to Architectural Periodicals, CINAHL, the Cochrane Library, Compendex, the Design & Applied Arts Index, PsycINFO, ProQuest Dissertations & Theses Global, PubMed, Scopus, and Web of Science. No restrictions were applied to years searched, but language was limited to English, Swedish, Danish, Norwegian, and French publications only.

***Criteria for inclusion were all types of peer-reviewed scientific papers which examined all health outcomes and experiences in women and their babies during labor and birth as well as shortly after birth***.

Study protocols were excluded as having a different research focus. The birthing room was defined as the room where the birth takes place. Unintended or incidental birth places, such as in an ambulance during transport, were excluded as the features of these spaces are too diverse to inform meaningful conclusions. Excluded were studies exploring all other persons other than the birthing mother and baby present in the birthing room; that is, partners, staff (midwives, assisting nurses, doctors), and doulas/birth companions.

**Figure 1.**
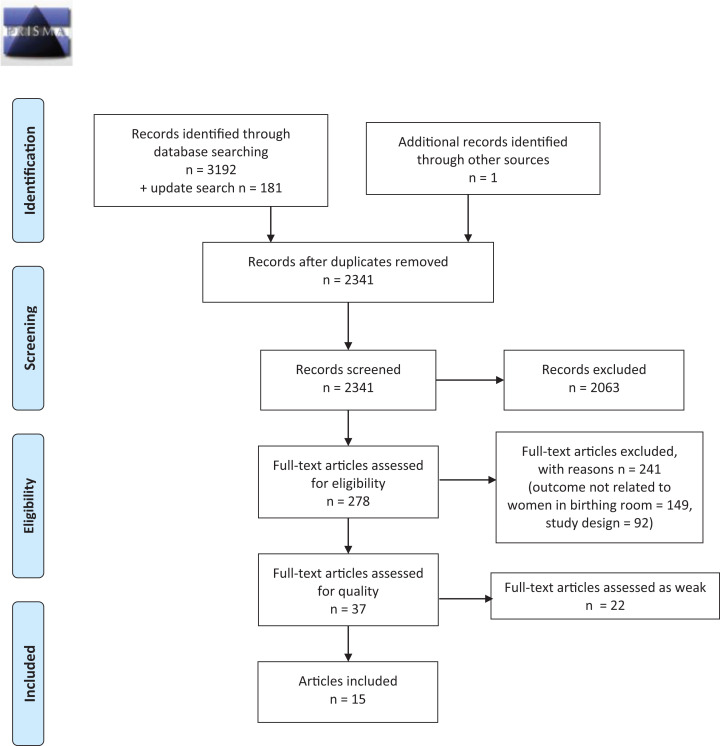
PRISMA flow diagram.

A search strategy was developed by the librarian authors (HS, EH) in collaboration with the other authors. The search strategy was reviewed for accuracy using the Peer Review of Electronic Search Strategies criteria (McGowan et al., 2016). Details of the search strategies are given in Supplemental File 1. Papers were screened and selected for inclusion or exclusion by team members working in pairs (CN & HW and LH & MB). Any disagreements were resolved by the author group together. Two reasons for exclusion were applied at the full-text screening stage. The first reason, study design, excluded all nonscientific articles and nonsystematic reviews. The second reason, outcome not related to women in birthing room, excluded outcomes and experiences in persons other than women in labor and birth and/or early after birth, such as staff, partners, and doulas/companions. However, one article describing couples’ mutual experiences and one article exploring support by partners/companions that created benefits for the woman were also included (Harte et al., 2016; Morison et al., 1998). The study selection process is illustrated in Figure 1, using Preferred Reporting Items for Systematic Reviews and Meta-Analyses (PRISMA) (Moher et al., 2009).

### Quality Assessment of Included Articles

Review team members working in pairs evaluated the quality of each article chosen for inclusion. The Effective Public Health Practice Project Tool (2009) was selected to assess the methodological quality of the quantitative studies. This tool includes components of study design and methods including selection and allocation bias, study design, confounding, blinding, data collection methods, and withdrawals/drop-outs. An overall quality rating was assigned to each article: if no weak ratings were given, the quality of the article was estimated to be “strong,” one weak rating categorized the article as “moderate,” and two or more weak ratings entailed the article being assessed as “weak” and consequently excluded from the analysis. Any disagreements regarding quality ratings were discussed and resolved by involving the other pair of reviewers. In addition, as one of the review team was an author of one of the articles, this study was assessed by the other pair of reviewers.

The quality of the qualitative studies was assessed using the Critical Appraisal Skills Programme [CASP] Qualitative Checklist (2017), which consists of 10 questions assessing different aspects of quality in qualitative studies. Although CASP does not grade the assessment, we graded each question’s answer on a scale of 0–2 points, with 2 representing highest quality. Consequently, each study could gain a score from 0 to 20 points, with 20 points representing the highest quality. A list of all articles which were excluded after quality assessment is given in Supplemental File 2.

## Results

### Process and Results of Search and Selection Strategy

In total, 3,373 records were identified. The initial database searches were performed on December 1, 2016, and resulted in 3,192 records. An update of the searches was performed on August 31, 2017, resulting in an additional 181 records. After removing duplicates, 2,341 unique records were screened by title and abstract and 2,063 records were excluded. The full texts of the remaining 278 articles were read, and 241 were excluded due to either study design or outcome not related to women in birthing rooms (for details, see Supplemental File 2), leaving 37 for quality assessment (see PRISMA flow diagram in Figure 1). After quality assessment, 22 additional articles were excluded due to having an overall “weak” rating, leaving 15 articles for analysis. Nine of these were quantitative studies: randomized controlled studies (4), a cohort study, controlled quasi-experimental studies (2), a descriptive comparative study, and an observational study (pre- and postintervention). The remaining six were qualitative studies using different methods: thematic interpretative reanalysis, ethnography (2), grounded theory, phenomenology, and secondary hermeneutic analysis. The studies had been conducted in six countries, mostly in high- and middle-income countries: the United States (5), Australia (4), Canada (2), Iran (2), China (1), and Sweden (1), between the years 1985 and 2017. Characteristics of the 15 articles are presented in Table 1.

**Table 1.** Characteristics and Quality Assessments of the 15 Studies Included in the Analysis.

First Author, Year of Publication	Rating EPHPP^a^ [CASP^b^]	Aim of Study	Study Design	Population	Sample Size	Country
Aburas (2017)	M	To examine the impact of presenting images of nature in the labor and delivery room on women’s experiences of labor and delivery	Randomized controlled trial	Pregnant women with intended vaginal birth	*n* = 50 (intervention group *n* = 24, control group *n* = 26)	USA
Hanson (2001)	[15]	To provide a fresh look at birth and allow for a fuller picture of the multiple dimensions of the experience that are derived from birth story data	A thematic reanalysis through theatrical interpretation	87 birth stories from women who gave birth at hospital or at home	*n* = 37 (primiparous *n* = 15, multiparous *n* = 22)	USA
Harper (1991)	M	To study the incidence, time of onset, severity, duration, temperature patterns, and relationship of various perinatal factors to the postpartum shivering phenomenon. To examine differences in temperature patterns and room temperature between parturients who shiver and those who do not	Cohort study	Women birthing through Lamaze technique	*n* = 50	USA
Harte (2016)	[18]	To explore inhibiting and facilitating design factors influencing childbirth supporters’ experiences	Ethnographic study	One case (a primiparous woman, her husband, her mother, two midwives, and two midwifery students)	*n* = 6	Australia
Hauck (2008)	[19]	To explore women’s experiences of using a Snoezelen room^c^ during labor	Grounded theory study	Women who had used a Snoezelen room during labor	*n* = 16 (primiparous *n* = 14, multiparous *n* = 2)	Australia
Hodnett (1989)	S	To investigate factors influencing the experience of control during childbirth among low-risk women choosing two different birth environments	A controlled quasi-experimental study	Women choosing hospital birth or home birth	*n* = 160 (hospital birth *n* = 80, home birth *n* = 80)	Canada
Hodnett (2009)	M	To examine the feasibility of a randomized trial and the acceptability of a modified labor room among women and their care providers	Randomized controlled trial	Pregnant women in labor and after birth	*n* = 62 (intervention group *n* = 31, control group *n* = 31)	Canada
Jia (2013)	M	To examine whether increasing the delivery room temperature results in increased temperature in premature newborns	Randomized controlled trial	Newborns ≤32 weeks’ gestation	*n* = 91 (intervention group *n* = 43, control group *n* = 48)	China
Lewis (2011)	M	To assess the effects of three interventions compared to control on thermoregulation in low birth weight infants	Quasi-experimental study	Infants weighing <1,500 g	*n* = 133	USA
Manesh (2015)	M	To determine the effect of a Snoezelen room on the duration of first and second stages of labor, labor pain intensity, and perineal injuries	Randomized controlled trial	Nulliparous women in labor	*n* = 100 (intervention group *n* = 50, control group *n* = 50)	Iran
Mondy (2016)	[17]	To explore and compare birth spaces with different domestic characteristics and examine how laboring women worked within these spaces during the labor process	Ethnographic study	Women who gave birth at hospitals or a birth center	*n* = 6 (hospitals *n* = 5, birth center *n* = 1)	Australia
Morison (1998)	[20]	To describe the experience of couples who had a home birth	Phenomenological approach study	Couples who had experienced home birth	*n* = 20 (couples *n* = 10)	Australia
Nilsson (2014)	[20]	To gain a deeper understanding of women’s negative experiences in the delivery room	Secondary hermeneutic analysis on three qualitative studies	Pregnant women with fear of childbirth and women with previous fear of childbirth	*n* = 21 (pregnant women *n* = 15, nonpregnant women *n* = 6)	Sweden
Pirdel (2009)	M	To examine the relationship between physical environmental factors and pain perception among parturient women	Descriptive comparative study	Women with vaginal birth	*n* = 600 (primiparous *n* = 300, multiparous *n* = 300)	Iran
Strobel (1985)	M	To examine the physiological and psychological effects of a humanized labor room environment on the labor process	Observational study, pre- and postintervention	Women with a vaginal birth	*n* = 197 (preintervention *n* = 92, 13 primipara, 79 multipara; postintervention *n* = 105, 32 primipara, 73 multipara)	USA

^a^ EPHPP = The Effective Public Health Practice Project quality assessment tool (W = weak, M = moderate, S = strong). ^b^CASP = Critical Appraisal Skills Programme (score 0–20 points, where 20 represents highest quality). ^c^The Snoezelen room is a concept whereby an indoor environment is created to provide comfort using controllable stimuli.

The results of the search process of literature dealing with how birthing room design influences maternal and neonate physical and emotional outcomes are presented according to the specific environmental factors highlighted in the assessed articles (Table 2).

**Table 2.** Findings: Influences of Birthing Room Design on Physical and Emotional Maternal and Neonate Outcomes.

First Author, Year of Publication	Key Findings Reported by Authors	Potential Design Factors Explaining the Outcome Suggested by Authors	Recommendations Made by Authors	Comments Made by Review Authors
**Snoezelen room** ^a^				
Hauck (2008)	The Snoezelen environment offers women: Distraction Relaxation Comfort Environmental control Choice of complementary therapies Safety in a homelike atmosphere	Polished floorboards, walls in soft earthy colors linking to birth and nature, comfortable furnishings, and soft rugs and ottomans. The main feature is the rotation wheel projector displaying wall patterns and fiber-optic lights with changing colors. An aquarium with tropical fish, relaxation music, and aromatherapy of the client’s choice complement the ambience of the room	Offer complementary therapies within the safety of a hospital environment. Support choice of relaxation music and aromatherapy	The Snoezelen room provides distraction, comfort, control, and safety
Manesh (2015)	Differences in pain intensity Visual Analogue Scale (VAS) between experimental and control groups before the intervention were not significant. After 3 hr, pain intensity was significantly higher among women in the control group compared to the intervention group (*p* = .01)	The Snoezelen room provided possibilities to walk, sit, and lie down, as well as an aquarium and projector with light and pictures on the wall, lavender essence, candles, and light music and sound. Both groups were given the possibility to walk, drink, and eat soft and sweet food	The distracting sensations in the Snoezelen room decreased the mother’s pain intensity, the length of labor, and the incidence of episiotomy	The Snoezelen room has positive effects on labor pain and the duration of the first stage of labor
**Room temperature, room equipment for babies**				
Harper (1991)	The mean birthing room temperature for shiverers was lower than for nonshiverers (*p* < .009), as was the mean recovery room temperature (*p* < .03)	Environmental temperature may play a heretofore unsuspected role in the postpartum shivering phenomenon	Missing	Room temperature impacts shivering in birthing women
Jia (2013)	Premature babies born in rooms with higher mean temperature (25.1°C) compared with those born in rooms with lower mean temperature (22.5°C) had a higher rectal temperature at admission to neonatal ward. This difference persisted after adjustment for birth weight and 5-min Apgar score	Increasing the birthing room temperature to that recommended by the World Health Organization decreases cold stress in premature newborns	Follow the World Health Organization recommendation of 25°C	Room temperature impacts heat loss in premature neonates
Lewis (2011)	Occlusive wrap, chemical mattress, and increased birthing room temperature together increased temperature at NICU admission in neonates <1,500 g (not significant)	Increased birthing room temperature	Using the polyethylene wrap, the chemical mattress, and increased birthing room temperatures to diminish hypothermia among low birth weight neonates is a modest financial investment which may affect mortality and morbidity rates, improving outcomes and lowering hospital costs	Means to avoid heat loss in low birth weight neonates
**Place for partner**				
Harte (2016)	Birth supporters experience “an unbelonging paradox” of being needed yet “in the way” during “tenuous nest-building” activities	In labor wards, supporters are inhibited from modifying the environment to create a familiar and safe space for the birthing woman Privacy needs, control, and choice-making possibilities are difficult to satisfy Technocratic environment conveys mixed messages Finding a place and purpose in the birth unit can be challenging for the supporter There is little support for the supporter’s physical needs	Sufficient space to accommodate multiple supporters Easily accessible storage space Aesthetically pleasing colors and images A family alcove near the entrance Medical equipment hidden Comfortable and movable furnishings Options to facilitate personal choice Readily available built-in physical supports Birth tubs Comfortable places to rest or sleep. Nourishing food and drink Easily accessible toilet facilities Posters or brochures for advice	A familiar environment supports and strengthens the companions and the birthing woman
**Sensory stimulation (nature, sounds, smells, visual)**				
Aburas (2017)	Nature images influence the labor experience positively in terms of higher patient-experienced care quality, lower heart rate in women (not significant), and higher Apgar score in neonates at 5 min (*p* = .05)	Different nature images displayed on a TV: trees, flowers, water, and other nature content	The results of this study emphasize the importance of incorporating nonpharmacological techniques in the labour room (e.g., nature images)	Presentations of nature image in the birthing room may have a positive impact on women
Hodnett (2009)	Women’s and practitioners’ qualitative evaluations of the ambient room were generally very positive Women in the ambient group were less likely to have artificial oxytocin during first or second stage labor (*p* = .03) 65% of women in the ambient room reported spending 50% less time in the standard labor bed, compared to 13% of women in standard rooms	Instead of a standard bed, a portable double-sized mattress with several large pillows was set up in a corner with the intent to allow the woman freedom in positioning, to permit close contact with support people, and to limit the routine use of continuous electronic fetal heart rate monitoring, and other technologies and equipment, thus facilitating upright positioning in labor. Dimmed lightning, DVD films of nature images on the wall, and a wide variety of music selections were available	The effect of this ambient room should be systematically evaluated in an adequately powered trial	Environmental modifications including the removal of standard bed and the addition of equipment promoting relaxation, mobility, and calm result in less need for artificial oxytocin
Strobel (1985)	No difference in length of labor between women in the humanized room, compared to women in the institutional room Women in the humanized room rated the labor experience as more pleasant than expected (*p* < .01), and the room as more desirable (*p* < .001)	Modifications to a more homelike, familiar setting; colored wallpaper and textile curtains, silk flower arrangements, and pictures	It is recommended that hospitals adapt the healthcare environment to a more humanized environment	An adapted labor room is perceived as more pleasant and desirable Differences in length of labor between primiparas and multiparas were not controlled for
Pirdel (2009)	Environmental factors were major predictors of pain in primiparas (*p* < .05) and multiparas (*p* < .05) Primiparas: The most important factors were a large number of patients in the labor room (70%) and restriction of movement and mobility (67%) Multiparas: The greatest stressors were related to noise in the labor ward (84%) and restriction of fluid intake (78%)	Of 20 environmental factors, noise was identified as an unpleasant factor for multiparas	A better understanding of pain perception in labour and birth requires that future studies focus on psychological factors that have different meaning to different women	Environmental factors affect pain perception. Noise is a stressor Unclear how the 20 environmental items were identified The environmental items were not specified; for instance, noise, crowded room, room lighting
**Familiarity, noninstitutionalized environment, institutionalized environment**				
Hodnett (1989)	Women giving birth at home had a greater degree of affiliation with caregivers and more freedom of exploration and self-expression compared to women at hospital who experienced more aversive stimuli. The home birth group had significantly higher levels of perceived control during childbirth The combination of expectations of control and type of childbirth setting accounted for 33.5% of the variance in perceived control scores	Environments differed in the extent to which they permitted freedom of movement and self-expression. Giving birth at home compared to hospital birth had advantages such as walks in a nearby park, showering or sitting in a warm tub of water, eating and drinking, music, TV, leaning over dining room furniture for support, and freely vocalizing their discomfort	The results give support for a model of person–environment interaction during child birth	The environment provides a level of control
Nilsson (2014)	The women’s experiences were interpreted as their being objects of surveillance in the delivery room, and they endured suffering related to care during childbirth. This involved experiences of feeling suppressed, unprotected, and lacking safety, and of midwives as uncaring Birth environments are understood as power structures, containing views of women’s birthing bodies as machines that have to be kept under surveillance	Features increasing the perception of surveillance in the physical environment, and technical equipment that limits women’s freedom to move during labor and birth; e.g., interventions such as fetal heart monitoring, induction and augmentation of labor. Environments that promote positive relationships between women and labor ward staff	Women must be offered not only medical safety but also emotional and existential safety in the labour room	The birthing room as a surveillance environment entails unnecessary suffering in women, creating fear of childbirth This calls for a reduction in the intrusion of technical devices and enhanced possibility for positive encounters between the women and the midwives and other staff
Hanson (2001)	Props such as various types of hospital equipment are often used to intervene in the birth process Hospital attire may be color-coded Some props/equipment assist the woman to use alternative positions. This equipment may be supplied by the birthing woman (Lamaze bag) or creatively used by the intrapartum nurse The hospital environment may be unfamiliar to the birthing woman	Continuous electronic fetal monitoring may result in the nurse/midwife spending less time with the woman, whereas the use of intermittent auscultation (via the pinard or a doptone) can result in a reorientation of the midwife/nurse’s role to remain with the woman	Equipment such as the pinard can enhance the possibilities for nurses/midwives to remain by the woman’s side	Unfamiliar environment may negatively distract birthing women
Morison (1998)	Couples giving birth at home adapted the physical environment and established support to create a positive birth environment	Homebirth entailed a process where a couple actively created an environment that enabled them to assume control and responsibility for the birth	Issues specific to the birth environment at home are relevant to parents’ possibilities to assume control and responsibility for the birth of their baby	Familiar environment affects perception of control and responsibility
Mondy (2016)	Giving birth at hospital made the women act and interact in a passive way, adapted to the space, and taking on the patient role Giving birth in a domestic birth environment (birthing center or home) made the women claim ownership of the space and express their identity. The design, furnishings, and semiotics of the space openly encouraged them to be active and creative and take ownership of the space	The features of domesticity within the birth setting may shape the experiences of laboring women and their care providers	The results can assist those who design, build, furnish, manage, access, and work in birthing services giving guidance on how homelike/familiar birth spaces should look in order to affect the outcomes and experiences of women and their families	Familiarity (a homelike environment) affects outcome and experiences

Note. NICU = Neonatal Intensive Care Unit; VAS = Visual Analogue Scale.

^a^ The Snoezelen room is a concept whereby an indoor environment is created to provide comfort using controllable stimuli.

A predesigned data extraction form, developed by the authors in relation to the study aim, was used to extract data on the articles’ characteristics and their results on how birthing room designs physically and emotionally influenced maternal and neonate outcomes. Data were first extracted independently by each member of the two review pairs and then checked for accuracy by the other reviewer. Next, joint discussions were conducted by the research team (including both review pairs) to analyze findings from the different articles. Five characteristics of birthing room design were derived: Snoezelen room, room temperature, place for partner, sensory stimulation, and familiarity, noninstitutionalized, institutionalized environment. The concept of Snoezelen, which was specifically explored in two of the studies, refers to the means whereby an indoor environment is created to provide comfort using controllable stimuli (Hauck et al., 2008; Manesh et al., 2015).

Despite the limited number of studies showing which specific aspects of birthing room design have an influence on maternal and neonate physical and emotional outcomes, four physical themes were more prominent and repeated than others: means of distraction, comfort, and relaxation; raising the birthing room temperature; features of familiarity; and diminishing a technocratic environment. These themes are presented individually below, but it should be noted that several of them seem to be closely related and interact with each other.

#### Means of distraction, comfort, and relaxation

Some of the studies stressed the importance of incorporating means of distraction, comfort, and relaxation in the design of the hospital environment, showing the resulting positive effects on labor and pain (Hauck et al., 2008; Manesh et al., 2015; Strobel, 1985), the woman’s heart rate, the neonate’s 5-min Apgar score (Aburas et al., 2017), and the need for artificial oxytocin (Hodnett et al., 2009). Methods of distraction integrated into the room included patterns or pictures projected on the wall via a rotating wheel projector and fiber-optic lights with changing colors (Hauck et al., 2008; Manesh et al., 2015), nature images chosen by the woman and displayed on a TV (Aburas et al., 2017) or via a DVD (Hodnett et al., 2009), an aquarium containing tropical fish (Hauck et al., 2008; Manesh et al., 2015), relaxation music and aromatherapy (e.g., lavender essence) of the woman’s choice, dimmed light or candlelight, and light music and sound (Aburas et al., 2017; Hauck et al., 2008; Hodnett et al., 2009; Manesh et al., 2015). Silk flower arrangements and pictures were other ways to support distraction, leading to the women’s experience of labor being more pleasant than expected (Strobel, 1985). One important observation was that not all sounds were helpful; noise in the labor room was identified as an unpleasant factor and a major predictor of pain for multiparas (Pirdel & Pirdel, 2009). See Table 1.

#### Raising the birthing room temperature

This was shown in controlled studies to have a significant impact on both the birthing woman and the neonate, measured as less postpartum shivering in women (Harper et al., 1991) and less heat loss in neonates (Jia et al., 2013; Lewis et al., 2011). To avoid heat loss among premature neonates, an increase of temperature of 2.6°C (from the usual 22.5°C to 25.1°C) seems to be needed (Jia et al., 2013). In addition to increased room temperature, the use of occlusive wraps and chemical mattresses was shown to be effective in diminishing heat loss among low birth weight neonates (Lewis et al., 2011).

#### Features of familiarity

These were shown to shorten the length of labor (Lewis et al., 2011) and to decrease labor pain intensity and the incidence of episiotomy (Manesh et al., 2015). A homelike environment is also of importance to allow the couple together to accomplish a feeling of respect and control (Morison et al., 1998), and for the partner to feel able to engage in securing a familiar and safe space for the birthing woman, to find their own purpose in the birth unit, and to have a place to rest and sleep (Harte et al., 2016). The inclusion of domestic features should be taken into account early in the design process, as a means to affect the outcomes and experiences of women and their families (Mondy et al., 2016). Design features that promote domesticity include polished floorboards, colored wallpaper or walls painted in soft earthy colors, textile curtains, birth tubs, comfortable furniture, and soft rugs and ottomans (Harte et al., 2016; Hauck et al., 2008; Strobel, 1985). Specifically, the replacement of the standard bed with a double-sized mattress and several large pillows can support the birthing woman in free positioning, which in turn promotes relaxation, mobility, and calm and leads to less need for artificial oxytocin (Hodnett et al., 2009). The amount of space also seems to influence the sense of domesticity, by giving the woman a free choice to walk around, sit or lie down, and eat or drink; this impacts the duration of labor and the experience of labor pain (Hauck et al., 2008; Manesh et al., 2015). A sufficient amount of space is also important for those supporting the women, providing them with a private space as well as a space for storage (Harte et al., 2016).

#### Diminishing a technocratic environment

This is important in order to support the woman’s freedom of movement and self-expression, to decrease unnecessary suffering due to fear of childbirth and to enhance the possibility for positive encounters between the women, the midwives, and other staff (Hodnett, 1989; Nilsson, 2014); to enable nest-building activities; and to avoid mixed messages between giving birth as a natural event or a high-tech incident (Harte et al., 2016). Possibilities to conceal medical equipment should be included in the design process, and the routine use of continuous electronic fetal heart rate monitoring and other technologies should be limited (Hodnett et al., 2009). For example, the use of continuous electronic fetal monitoring has been shown to lead to a risk that the midwife/nurse will spend less time in the birthing room, which in turn may distract the birthing woman in a negative way (Hanson et al., 2001).

## Discussion

The result of this literature review on how birthing room design influences maternal and neonate physical and emotional outcomes revealed four prominent physical themes that affect women and their babies: means of distraction, comfort, and relaxation; raising the birthing room temperature; features of familiarity; and diminishing a technocratic environment.

The findings of this review also show that research into the birthing environment is far too limited, and there is a need for high-quality scientific studies on the effect of the birthing room itself. Existing research is fragmented, describing only some parts of the effect of environments surrounding labor and birth. The results indicate knowledge gaps in several different dimensions of birthing room design: for example, the role of distraction, how and when is it beneficial to women (Aburas et al., 2017; Hauck et al., 2008; Hodnett et al., 2009; Manesh et al., 2015), and when is it disturbing (Pirdel & Pirdel, 2009). Feelings of familiarity gave positive effects (Harte et al., 2016; Manesh et al., 2015; Mondy et al., 2016; Morison et al., 1998; Strobel, 1985), yet it is unclear what is included in the term familiarity. Diminishing a technocratic environment has been shown to have effects, but how should technologies be used to not become a hinder to women’s freedom to move during labor and birth and not obstruct midwives possibilities to connect and create a relationship to birthing women (Hanson et al., 2001; Harte et al., 2016; Hodnett, 1989; Nilsson, 2014). Some women feel safer with medical equipment, others do not. How can we create birthing rooms that suit all women? How can a sense of integrity be designed (Nilsson, 2014)? The results in the review by Setola et al. (2019) give complementary information to the gaps of knowledge, for example, how an interface to the birthing room can work as a filter marking the privacy, and thereby promote the integrity of the birthing room. However, the effect of such interventions in the birthing room environment needs to be further evaluated.

***…research into the birthing environment is far too limited, and there is a need for high-quality scientific studies on the effect of the birthing room***.

Moreover, the findings also point to a favoring of a “birth territory ideology”; a theory in which the birthing environment is described as a safe space, where women’s power to give birth physiologically is promoted and their privacy is protected (Foureur, 2008; Stenglin & Foureur, 2013). This theory is in contrast with the more technocratic ideology practiced in high-tech environments (Stenglin & Foureur, 2013).

The findings in our review give an indication that hospital-based labor environments may need to be adjusted to better fit women’s needs during labor and birth. Several of the physical aspects presented in the results are environmental factors that promote the release of endogenous oxytocin in women, with positive effects on the duration of first stage of labor, the experience of labor pain (Hauck et al., 2008; Manesh et al., 2015; Strobel, 1985), and lower heart rate in women (Aburas et al., 2017), which accordingly entails fewer interventions with artificial oxytocin (Hodnett et al., 2009). To be able to create safe birth spaces for women, knowledge of how the oxytocin system works is vital (Foureur, 2008). Oxytocin is stimulated by pleasant sounds, scenes of nature, and relaxing activities (Foureur, 2008); all described as *means of distraction, comfort, and relaxation* (Aburas et al., 2017; Hauck et al., 2008; Hodnett et al., 2009; Manesh et al., 2015; Strobel, 1985). In contrast, disturbing noise is a predictor for pain (Pirdel & Pirdel, 2009), in line with research on factors that can inhibit the release of oxytocin in women (Foureur, 2008).

The physical characteristics *features of familiarity* and *diminishing a technocratic environment* can be related to the place of birth. Many women in high- and middle-income countries are expected to give birth at high-tech hospital-based labor wards, in environments that are often functional, impersonal, and clinical (Newnham et al., 2018). The impact of a technocratic birth ideology is evident in many hospital labor wards, entailing medical intervention and a high level of surveillance of the birthing woman (Newnham et al., 2018; Nilsson, 2014; Stenglin & Foureur, 2013). A technocratic ideology also influences the design of hospital buildings and of the physical birthing environment as intensive care units with blinking lights, alarms, and monitors. Such environments can be related to medical interventions indirectly, through women and health professionals’ behavior, experiences, and practices during labor and birth (Setola et al., 2019). The theory of birth territory developed by Fahy and colleagues (2011) points out the necessity of creating birthing environments as a sanctum for birthing women rather than a surveillance environment. This theory can be related to some of the findings in the present review as promoting nest-building activities and viewing birth as a natural life event (Harte et al., 2016), in contrast to the reports of fear of childbirth among women (Nilsson, 2014). Features of familiarity include the diminishing and covering of medical equipment, as well as making a place for partners and birth companions to create a familiar and safe atmosphere for the woman and themselves (Harte et al., 2016; Morison et al., 1998).

### Limitations

Leaning on the definition of a systematic review from Grant and Booth (2009), we aimed to find all known knowledge on the topic irrespective of study design and discipline. We searched for studies in 10 databases within midwifery, nursing, medicine, and architecture covering the potential research field; however, we had to limit the search to the five languages in which the research team was fluent. We found relatively few articles presenting specific elements of birth room design that physically and emotionally influenced maternal and neonate outcomes. The methodological quality of the articles varied, and we had to exclude more than half of the eligible articles due to weak quality ratings. All these circumstances may have affected the results of the review. Given that both quantitative and qualitative articles were included in the review, the studied populations were heterogenic in terms of characteristics and sample sizes, and different measurement tools and data analysis strategies were used, the findings of this review should be taken with particular caution. These factors meant that a meta-analysis could not be performed; instead, a narrative analysis was accomplished, revealing four physical themes that were shown to influence maternal and neonate physical and emotional outcomes. The extent to which this study’s result can be generalizable to other birthing room environments have to be assessed by the single readers of this article, who can relate the findings to their specific birth context and decide whether or not they are applicable.

## Conclusions

The results of this review demonstrate limited evidence on birthing room design that promotes health in birthing women and their babies, particularly in hospital-based labor wards. Given that many women are expected to give birth at labor wards in hospitals, it is surprising that the evidence on how such environments affect the health of women and their babies is insufficient. Four prominent physical themes in birthing room environments have however been identified to positively influence maternal and neonate physical and emotional outcomes: (1) means of distraction, comfort, and relaxation; (2) raising the birthing room temperature; (3) features of familiarity; and (4) diminishing a technocratic environment.

***Given that many women are expected to give birth at labor wards in hospitals, it is surprising that the evidence on how such environments affect the health of women and their babies is insufficient***.

### Recommendations for Future Research

The results indicate knowledge gaps in several different dimensions of birthing room design. The evidence on how birthing environments affect outcomes of labor and birth in women and neonates is all too limited. There is crucial need for more research in this field.

## Implications for Practice

- Birthing room environments including means of distraction, comfort, and relaxation; raising the birthing room temperature; and features of familiarity may have positive effects on outcomes of labor and birth
- Diminishing a technocratic birthing environment can positively affect outcomes of labor and birth
- The results of this review can be used to guide the design of birthing room environments in order to improve patient safety and well-being

## Supplemental Material

Supplemental Material, sj-docx-1-her-10.1177_1937586720903689 - Effects of Birthing Room Design on Maternal and Neonate Outcomes: A Systematic Review Click here for additional data file. Supplemental Material, sj-docx-1-her-10.1177_1937586720903689 for Effects of Birthing Room Design on Maternal and Neonate Outcomes: A Systematic Review by Christina Nilsson, Helle Wijk, Lina Höglund, Helen Sjöblom, Eva Hessman and Marie Berg in HERD: Health Environments Research & Design Journal

Supplemental Material, sj-docx-2-her-10.1177_1937586720903689 - Effects of Birthing Room Design on Maternal and Neonate Outcomes: A Systematic Review Click here for additional data file. Supplemental Material, sj-docx-2-her-10.1177_1937586720903689 for Effects of Birthing Room Design on Maternal and Neonate Outcomes: A Systematic Review by Christina Nilsson, Helle Wijk, Lina Höglund, Helen Sjöblom, Eva Hessman and Marie Berg in HERD: Health Environments Research & Design Journal
