# Supplementary material for: Effects of Birthing Room Design on Maternal and Neonate Outcomes: A Systematic Review
Source: HERD. 2020 Feb 20;13(3):198–214. doi: 10.1177/1937586720903689 (PMC7364772; doi:10.1177/1937586720903689)
Supplement: Supplemental Material, sj-docx-1-her-10.1177_1937586720903689 - Effects of Birthing Room Design on Maternal and Neonate Outcomes: A Systematic Review [file sj-docx-1-her-10.1177_1937586720903689.docx]

**Search strategy**All databases were searched from inception date. The initial search was made December 1 2016.

**PubMed**((design* OR facilit* OR interior OR environment* OR furnish* OR equipment OR snoezelen OR salutogen*) AND ("delivery rooms" OR "delivery room" OR "birth center" OR "birth centers" OR "birth centre" OR "birth centres" OR "birth room" OR "birth rooms" OR "birthing room" OR "birthing rooms" OR “labor room” OR “labour room” OR “labor rooms” OR “labour rooms” OR "birth environment" OR "birth environments" OR “birth unit” OR “birth units”)) OR (("Hospital Design and Construction"[Mesh] OR "Interior Design and Furnishings"[Mesh] OR "Hospital Planning"[Mesh] OR "Environment Design"[Mesh] OR "Health Facility Environment"[Mesh]) AND ("Parturition"[Mesh] OR "Labor, Obstetric"[Mesh] OR "Delivery Rooms"[Mesh]))

Limit language English, Swedish, Danish, Norwegian, French
n=1258

**Cinahl**S1
 ( "delivery rooms" OR "delivery room" OR "birth center" OR "birth centers" OR "birth centre" OR "birth centres" OR "birth room" OR "birth rooms" OR "birthing room" OR "birthing rooms" OR “labor room” OR “labour room” OR “labor rooms” OR “labour rooms” OR "birth environment" OR "birth environments" OR “birth unit” OR “birth units” ) AND ( design* OR facilit* OR interior OR environment* OR furnish* OR equipment OR snoezelen OR salutogen* ) 

S2
((MH "Hospital Design and Construction+") OR (MH "Interior Design and Furnishings+") OR (MH "Hospital Planning+") OR (MH "Environment Design+") OR (MH "Health Facility Environment+")) AND ((MH "Parturition+") OR (MH "Labor, Obstetric+") OR (MH "Delivery Rooms+")) 

S1 OR S2

Limit language English, Swedish, Danish, Norwegian, French
Exclude Medline records
n= 211

**Cochrane**Search all text
("delivery rooms" or "delivery room" or "birth center" or "birth centers" or "birth centre" or "birth centres" or "birth room" or "birth rooms" or "birthing room" or "birthing rooms" or "labor room" or "labour room" or "labor rooms" or "labour rooms" or "birth environment" or "birth environments" or "birth unit" or "birth units") AND (design* or facilit* or interior or environment* or furnish* or equipment or snoezelen or salutogen*)

n= 287

**Web of Science**S1
TOPIC: ("delivery rooms" OR "delivery room" OR "birth center" OR "birth centers" OR "birth centre" OR "birth centres" OR "birth room" OR "birth rooms" OR "birthing room" OR "birthing rooms" OR “labor room” OR “labour room” OR “labor rooms” OR “labour rooms” OR "birth environment" OR "birth environments" OR “birth unit” OR “birth units”)

**S2**TOPIC: (design* OR facilit* OR interior OR environment* OR furnish* OR equipment OR snoezelen OR salutogen*)

S1 AND S2

Limit language English, Swedish, Danish, Norwegian, French
n= 731

**PSYCINFO**((SU.EXACT.EXPLODE("Labor (Childbirth)") OR SU.EXACT.EXPLODE("Birth")) AND (SU.EXACT.EXPLODE("Hospital Environment") OR SU.EXACT.EXPLODE("Interior Design"))) OR (("delivery rooms" OR "delivery room" OR "birth center" OR "birth centers" OR "birth centre" OR "birth centres" OR "birth room" OR "birth rooms" OR "birthing room" OR "birthing rooms" OR "labor room" OR "labour room" OR "labor rooms" OR "labour rooms" OR "birth environment" OR "birth environments" OR "birth unit" OR "birth units") AND (design OR facilit* OR interior OR environment* OR furnish* OR equipment OR snoezelen OR salutogen*))

Limit language English, Swedish, Danish, Norwegian, French
n=145

**Avery Index to Architectural Periodicals**"delivery rooms" OR "delivery room" OR "birth center" OR "birth centers" OR "birth centre" OR "birth centres" OR "birth room" OR "birth rooms" OR "birthing room" OR "birthing rooms" OR “labor room” OR “labour room” OR “labor rooms” OR “labour rooms” OR "birth environment" OR "birth environments" OR “birth unit” OR “birth units”

Limit language English, Swedish, Danish, Norwegian, French
n=11

**Design & Applied Arts Index (DAAI)**
"delivery rooms" OR "delivery room" OR "birth center" OR "birth centers" OR "birth centre" OR "birth centres" OR "birth room" OR "birth rooms" OR "birthing room" OR "birthing rooms" OR “labor room” OR “labour room” OR “labor rooms” OR “labour rooms” OR "birth environment" OR "birth environments" OR “birth unit” OR “birth units”

Limit language English, Swedish, Danish, Norwegian, French
n=5

**Compendex**"delivery rooms" OR "delivery room" OR "birth center" OR "birth centers" OR "birth centre" OR "birth centres" OR "birth room" OR "birth rooms" OR "birthing room" OR "birthing rooms" OR "labor room" OR "labour room" OR "labor rooms" OR "labour rooms" OR "birth environment" OR "birth environments" OR "birth unit" OR "birth units"

Limit language English, Swedish, Danish, Norwegian, French
n= 47

**Scopus**TITLE-ABS-KEY ( "delivery rooms"  OR  "delivery room"  OR  "birth center"  OR  "birth centers"  OR  "birth centre"  OR  "birth centres"  OR  "birth room"  OR  "birth rooms"  OR  "birthing room"  OR  "birthing rooms"  OR  "labor room"  OR  "labour room"  OR  "labor rooms"  OR  "labour rooms"  OR  "birth environment"  OR  "birth environments"  OR  "birth unit"  OR  "birth units" )  AND  TITLE-ABS-KEY ( design*  OR  facilit*  OR  interior  OR  environment*  OR  furnish*  OR  equipment  OR  snoezelen  OR  salutogen* )  AND NOT  INDEX ( medline ) 

Limit language English, Swedish, Danish, Norwegian, French
n= 384

**ProQuest Dissertations & Theses Global**all("delivery rooms" OR "delivery room" OR "birth center" OR "birth centers" OR "birth centre" OR "birth centres" OR "birth room" OR "birth rooms" OR "birthing room" OR "birthing rooms" OR “labor room” OR “labour room” OR “labor rooms” OR “labour rooms” OR "birth environment" OR "birth environments" OR “birth unit” OR “birth units”)
AND all(design* OR facilit* OR interior OR environment* OR furnish* OR equipment OR snoezelen OR salutogen*)

Limit language English, Swedish, Danish, Norwegian, French
n=106
