# Supplementary material for: Effects of Birthing Room Design on Maternal and Neonate Outcomes: A Systematic Review
Source: HERD. 2020 Feb 20;13(3):198–214. doi: 10.1177/1937586720903689 (PMC7364772; doi:10.1177/1937586720903689)
Supplement: Supplemental Material, sj-docx-2-her-10.1177_1937586720903689 - Effects of Birthing Room Design on Maternal and Neonate Outcomes: A Systematic Review [file sj-docx-2-her-10.1177_1937586720903689.docx]

**Reasons for exclusion fulltext**

| Reason for exclusion | *n* |
| --- | --- |
| Study design | 92 |
| Outcome not related to women in birthing room | 149 |
| Total | *241* |

**Excluded papers with reason**

**Study design**

Delivery (obstetric) departments - functions, spatial programs, layouts (Swedish). (1974). *Spri Rad, 0*(19).

Sterile delivery room gives birth to homelike setting. (1979). *Contract, 21*(2), 60-61.

Home-like birthing room conceals medical equipment. (1984). *Contract, 26*(2), 92-93.

Create healing environment without music therapist. (1999). *Patient Focus Care Satisf, 7*(3), 28-30.

Building for a better birth. (2003). *Scottish Nurse, 7*(10), 7-7.

NCT call for improvements to the birth environment. (2003). *Positive Health, 0*(89), 8-8.

NCT calls for better birthing facilities: poor birth environments could reduce 'normal' births...National Childbirth Trust. (2003). *Practising Midwife, 6*(7), 8-8.

Creative arts space: birthing units: new solutions for products and spaces. (2011). *Women & Birth, 24*, S52-S52.

Update on: positions for labour and birth. (2012). *Essentially MIDIRS, 3*(2), 38-42.

Ahrentzen, S. (1986). Birth settings: a perspective on our progress. *Women and environments, 8*(1), 16-19.

Allgaier, A. (1978). Alternative birth centers offer family-centered care. *Hospitals, 52*(24), 97-98, 100, 103-104 passim.

Bajo, K. (1987). Obstetrical design trends reflect move toward personal touch. *Mich Hosp, 23*(8), 37-41.

Bajo, K., & Shidaker, T. (1984). Innovations in obstetric design that meet professional and consumer demands. *Hosp Admin Curr, 28*(2), 7-12.

Bishop, E., Giannasio, B., & Gavin, C. (2011). *Passage Rights: Reclaiming the Rite of Passage of Birth at Pier A The Methodology and Theoretical Underpinnings for the Transformation of New York's Historic Pier A into a Freestanding Birthing Center and Multi-Use Commercial Space.* (1506288).

Bock, S. F., & Brengman, S. L. (1986). A delivery room you can set up anywhere. *Rn, 49*(4), 28-30.

Bond, L., & Nelki, J. (1995). Positions in labour: a plea for flexibility. *Mod Midwife, 5*(2), 19-22.

Burmahl, B. (2000). Facility profile. Designers deliver spacious maternity center. Utah Valley Regional Medical Center, Provo, Utah. *Health Facil Manage, 13*(2), 14-15.

Carlisle, D. (1989). Midwifery: home-from-home. *Nurs Stand, 3*(42), 28.

Christensson, K., Lindgren, H. E., Radestad, I. J., & Hildingsson, I. M. (2008). Outcome of planned home births compared to hospital births in Sweden between 1992 and 2004. A population-based register study. *Acta Obstet Gynecol Scand, 87*(7), 751-759.

Cook, T. R., & Cook, L. F. (1977). Alternative birth environments: best of home, hospital combined. *Hosp Admin Curr, 21*(4), 13-18.

Davidsen, J. (1990). Birthing center. A hospitable hospital room by Loebl Schlossman and Hackl. *Inter Des, 61*(15), 190-193.

Davis, D., Fenwick, J., Forbes, I., Foureur, M., Leap, N., Homer, C. S. E., & Iedema, R. (2010). The relationship between birth unit design and safe, satisfying birth: Developing a hypothetical model. *Midwifery, 26*(5), 520-525.

Dawson, J., Morley, C., & Davis, P. (2016). Primary outcomes for delivery room randomised trials-a review. Journal of paediatrics and child health. Conference: 20th annual meeting of the perinatal society of australia and new zealand, PSANZ 2016. Australia. Conference start: 20160522. Conference end: 20160525, 52, 3-4

Deakin, C. (2000). Care of newborn infants in delivery room. Two clarifications. *Bmj, 320*(7239), 937.

DeVries, R. G. (1979). Responding to consumer demand: a study of alternative birth centers. *Hosp Prog, 60*(10), 48-51, 68.

Edwards, N. (2003). Birth environments. *Midwifery Matters, 0*(99), 17-21.

Finer, N., Van Marter, L. J., Ramos, C., White, R., Martin, G., Ramanathan, R., . . . Bhatt, D. R. (2007). Transitional hypothermia in preterm newborns. *J Perinatol, 27*, S45-47.

Finer, N., Van Marter, L. J., Ramos, C., White, R., Martin, G., Ramanathan, R., . . . Bhatt, D. R. (2010). Transitional hypothermia in preterm newborns. *Adv Neonatal Care, 10*(5), S15-17.

Finigan, V., & Chadderton, D. (2015). Facilitate birth in water. *Midwives, 18*(3), 32-34.

Fonty, B., & Rydlowski, M. (1980). FRANSKA TILL MARIE [Psychological environment of delivery. Presence of the father in the labor room]. *J Gynecol Obstet Biol Reprod (Paris), 9*(1), 135.

Foureur, M., Homer, C., Davis, D., & Hammond, A. (2013). The hospital birth environment: Impacts on midwifery practice during labour and birth. *Women & Birth, 26*, S8-S8.

Foureur, M., Stewart, S., Sheehan, A., & Harte, J. D. (2013). CONFERENCE PROCEEDING The Birth Unit Design's influence on women's birth supporters. *Women & Birth, 26*, S28-29.

Fox, H. E. (1983). Renovations of an obstetrics suite: Regional Center for Tertiary Perinatal Care of Presbyterian Hospital in the City of New York. *Clin Perinatol, 10*(1), 167-173.

Gambito, K. (2006). Should videos and TV cameras be allowed at births? Pro. *MCN Am J Matern Child Nurs, 31*(1), 8.

Gaskie, M. F. (1990). Making special care special. Lake Pavilion/Family Birth Center, Baptist Hospital of Miami. *Archit Rec, 178*(7), 98-101.

Gerfen, K. (2005). It takes a (birthing) village. *Architecture, 94*(1), 12.

Gowers, D. (2002). Crowborough Birthing Centre: the pursuit of excellence. *MIDIRS Midwifery Digest, 12*(2), 264-268.

Grad, R. K. (1979). Breaking ground for a birthing room. *MCN Am J Matern Child Nurs, 4*(4), 245-249.

Gutteridge, K. (2014). The multisensory approach to birth and aromatherapy. *Pract Midwife, 17*(5), 26-29.

Gutteridge, K. (2016). How to... build and develop a birth centre. *Midwives, 19*(2), 36-37.

Hardy, C. T., & Ekbladh, L. (1978). Hospital meets patient demand for 'home-style' childbirth. *Hospitals, 52*(5), 73-74, 79-80.

Harelv-Lam, B., & Lucey, N. (2004). An integrated midwifery led birthing centre -- creating a successful birth centre within a hospital. *MIDIRS Midwifery Digest, 14*(1), S20-22.

Hodnett, E. D. (1984). *The effects of person–environment interactions on selected childbirth outcomes of women having home and hospital births.* (0).

Homer, C., Foureur, M., Catling-Paull, C., & Sheehy, A. (2011). Examining the content validity of the birthing unit design spatial evaluation tool within a woman-centered framework. *J Midwifery Womens Health, 56*(5), 494-502.

Hughes, M. (1981). The "birthing room" alternative. *Dimens Health Serv, 58*(6), 32-33.

Humenick, S. S. (2000). Birth environments. *J Perinat Educ, 9*(2), vi-vii.

Hunsberger, W. (1986). Canadian birthing rooms and centers: a status report. *NAACOG Newsl, 13*(5), 3, 8.

Inestam, E. (1989). [Sarasota Birthing House, Florida]. *Jordemodern, 102*(7), 305-306.

Jeffreys, P. (2000). Feng Shui for the health sector: harmonious buildings, healthier people. *Complement Ther Nurs Midwifery, 6*(2), 61-65.

Jones, O. (1997). Setting up a midwife managed birth centre. *MIDIRS Midwifery Digest, 7*(4), 514-516.

Kameyama, M. (2008). Influences by sound and light to mind and body during delivery, and future of childbirth environment. *Journal of Light and Visual Environment, 32*(2), 214-217.

Kanto, W. P., Jr., & Calvert, L. J. (1977). Neonatal resuscitation. *Am Fam Physician, 16*(6), 76-84.

Kennedy, H. P., & Neumann, Y. (2010). Homestyle midwifery: Lessons learned on bringing home to the hospital birth setting. *Journal of Midwifery & Women's Health, 55*(3), 273-276.

Kerner, J., & Ferris, C. B. (1978). An alternative birth center in a community teaching hospital. *Obstet Gynecol, 51*(3), 371-373.

Lam, J. (2014). Happy birthday: [Toronto, Ontario]. *Canadian architect, 59*(9), 29-31.

Lazarus, E. S. (1994). WHAT DO WOMEN WANT - ISSUES OF CHOICE, CONTROL, AND CLASS IN PREGNANCY AND CHILDBIRTH. *Medical Anthropology Quarterly, 8*(1), 25-46.

Leap, N., Homer, C. S. E., Foureur, M. J., Davis, D. L., & Forbes, I. F. (2010). Developing the Birth Unit Design Spatial Evaluation Tool (BUDSET) in Australia: A Qualitative Study. *Herd-Health Environments Research & Design Journal, 3*(4), 43-57.

Leap, N., Homer, C. S. E., Foureur, M. J., Davis, D. L., & Forbes, I. F. (2011). Testing the Birth Unit Design Spatial Evaluation Tool (BUDSET) in Australia: A Pilot Study. *Herd-Health Environments Research & Design Journal, 4*(2), 36-60.

Learner, S. (2005). Home comforts in labour room deliver more relaxed birthing. *Nursing Standard, 19*(41), 9-9.

Mahomed, K., Lawson, M., Hofmeyr, G., Nikodem, V., Gulmezoglu, A., & Walt, L. (1995). Companionship to modify the clinical birth environment: no measurable effect on stress hormone levels. *Journal of obstetrics and gynaecology, 15*, 178-181.

Maloni, J. A. (1980). The birthing room: some insights into parents' experiences. *MCN Am J Matern Child Nurs, 5*(5), 314-319.

Mance, M. J. (2008). Keeping infants warm: challenges of hypothermia. *Adv Neonatal Care, 8*(1), 6-12.

McQueen, R. J. (1966). The obstetrical suite takes on new dimensions. *Hospitals, 40*(9), 93-97.

Mogensen, K., Abdullahi, L., Kongsgaard, E., Sass, L., & Bock, J. (1990). [The significance of the environment for delivery at a special department]. *Ugeskrift for laeger, 152*(11), 732-734.

Newburn, M. (2003). Culture, control and the birth environment. *Pract Midwife, 6*(8), 20-25.

Newburn, M. (2006). Birth centres offer the best environment for normal birth. *British Journal of Midwifery, 14*(2), 84-85.

Newburn, M. (2006). Curtains for the old delivery suite. *Pract Midwife, 9*(1), 12-14.

Nikodem, C. (2002). Review: a home-like birth environment has beneficial effects on labor and delivery. *ACP J Club, 137*(1), 29.

O'Brien, M., Fellows, E., & Checketts, J. (1982). Hospital design: split in the nucleus. *Health Soc Serv J, 92*(4792), 473-476.

Page, L. (2002). Building for a better birth. *British Journal of Midwifery, 10*(9), 536-538.

Page, L. (2006). An ideal birth environment? The right facilities and support for women. *British Journal of Midwifery, 14*(1), 46-46.

Pandolfo, B., & Verghese, G. (2011). CONFERENCE PROCEEDING Plenary 11: Design briefs for birthing units are not all black and white. *Women & Birth, 24*, S33-34.

Phillips, R. (2017). Healthy Measures. Canadian Interiors, 54(3), 45-49.
Quayle, C. (1997). Mercy Hospital creates an intimate space for moms and newborns. *Health Facil Manage, 10*(1), 18-19.
Ralston, R. (1998). Aiming for excellence. Setting up woman-centred care at Ayrshire Central Maternity Hospital. *Pract Midwife, 1*(7), 50-52.
Robertson, A. F. (1971). Are radiant heaters safe in the delivery room? *Pediatrics, 47*(5), 955-956.
Rooks, J. P., & Ernst, E. K. (1990). Outcomes of care in Birth Centers. *Birth, 17*(4), 234.
Rosenberg, L., Milliken, R. A., & Milliken, G. M. (1972). A queuing theory model for the prediction of delivery room utilization. *Am J Obstet Gynecol, 114*(5), 691-699.
Rothman, B. K. (1983). Anatomy of a compromise: nurse-midwifery and the rise of the birth center. *J Nurse Midwifery, 28*(4), 3-7.
Scott, G. A. (2010). Emergency cesarean delivery in the labor and delivery room. *Surgical Technologist, 42*(8), 352-359.
Sheehy, A., Foureur, M., Homer, C., & Catling‐Paull, C. (2011). Examining the content validity of the birthing unit design spatial evaluation tool within a woman‐centered framework. *Journal of Midwifery & Women's Health, 56*(5), 494-502.
Stark, M. A., Zwelling, E., & Remynse, M. (2016). Importance of the Birth Environment to Support Physiologic Birth. *J Obstet Gynecol Neonatal Nurs, 45*(2), 285-294.
Stevens, N. R., & Hamilton, N. A. (2011). Perceived Control and Maternal Satisfaction with the Childbirth Experience. *3468861*, 147.
Stichler, J. F. (2007). Is your hospital hospitable? How physical environment influences patient safety. *Nurs Womens Health, 11*(5), 506-511.
Stockton, A. (2009). Birth space, safe place: environment, people and attitudes to pain during labour. *AIMS Journal, 21*(1), 9-10.
Stringer, M., & Miesnik, S. R. (2002). Technology in the birthing room. *Nurs Clin North Am, 37*(4), 781-793.
Sudbay, B. J., & Good, S. (1990). Labor environments and anxiety levels of primigravidas. *1343161*, 81.
Swayze, S. C. (1999). Labor and delivery beds. *Nursing, 29*(5), 74.
Sweet, A. Y. (1972). Radiant heaters and other hazards in the delivery room. *Pediatrics, 49*(1), 144-145.
Tetlow, K. (1992). Gentle discovery: Jain Malkin documents the criteria for a successful birthing center. *Interiors, 151*(12), 64-67.
Walsh, D. (2010). Birth Environment *Essential Midwifery Practice: Intrapartum Care* (Vol. 0, pp. 45-61).
Worth, J. (1999). Neo-natal sensitization to latex: A medical hypothesis. *Journal of Nutritional and Environmental Medicine, 9*(4), 305-312.

**Outcome not related to women in birthing room#**

# Outcome not related to the inclusion criteria: 1) maternal and/or infant health outcome (physical and emotional), 2) the definition of the birthing room.

Althabe, F., Fernández, A., Escobedo, M., Guinsburg, R., Aguilar, A., Szyld, E., . . . Saker, F. (2016). Risk factors for advanced resuscitation in term and near-term infants: A case-control study. *Archives of Disease in Childhood, 0*.

Armstrong, T. S., & Johnston, I. G. (2000). Which women want food during labour?: results of an audit in a Scottish DGH. *Health Bull (Edinb), 58*(2), 141-144.

Arya, S., Kapil, A., & Dadhich, A. (2014). Exploring the Pathogens Present at the Patient Care Equipments & Supplies to Sensitise the Health Care Workers for Preventing Health Care-Associated Infections among In-Patients. *Nurs J India, 105*(6), 283-286.

Atwood, J. R., & Lehrman, E.-J. (1988). *A theoretical framework for nurse-midwifery practice.* (8905798).

Bancalari, E. (1975). Resuscitation of the newborn. *Postgrad Med, 57*(3), 89-92.

Beard, R. W., & Capel, K. (1985). A better environment for women in labour. *Lancet, 2*(8463), 1059.

Birch, S., O'Brien-Pallas, L., & Murphy, G. T. (2001). Workforce planning and workplace management. *International Nursing Perspectives, 1*(2), 55-65.

Bissinger, R. L., & Annibale, D. J. (2010). Thermoregulation in very low-birth-weight infants during the golden hour: results and implications. *Adv Neonatal Care, 10*(5), 230-238.

Bracke, P., & Christiaens, W. (2009). Place of birth and satisfaction with childbirth in Belgium and the Netherlands. *Midwifery, 25*(2), e11-19.

Brent, R. L., McCullough, L. B., Arabin, B., Chervenak, F. A., Levene, M. I., Grunebaum, A., & Sapra, K. J. (2014). Early and total neonatal mortality in relation to birth setting in the United States, 2006-2009. *Am J Obstet Gynecol, 211*(4), 390.e391-397.

Broussard, A. B., & Lane, P. L. (1995). DISS Self-efficacy for childbirth: A qualitative study of pregnant women planning homebirth. *9542137*, 182.

Brown, Z. A., Clark, J. M., & Jung, A. L. (1976). Resuscitation equipment board for nurseries and delivery rooms. *Jama, 236*(21), 2427-2428.

Bunting, B., Kernohan, G., Sinclair, M., & Hatamleh, R. (2013). Birth memories of Jordanian women: Findings from qualitative data. *Journal of Research in Nursing, 18*(4), 235-244.

Cavallin, F., Zanardo, V., Trevisanuto, D., Doglioni, N., Coretti, I., & Udilano, A. (2011). Effective temperature under radiant infant warmer: does the device make a difference? *Resuscitation, 82*(6), 720-723.

Chabernaud, J. L. (2005). [Neonatal resuscitation in delivery room: new advances]. *Arch Pediatr, 12*(4), 477-490.

Clarke, P., Bowcock, M., & Gales, P. (2007). Development of an Integrated Care Pathway for natural birth. *British Journal of Midwifery, 15*(1), 12-15 14p.

Collins, C. T., Crowther, C. A., Smith, C. A., & Levett, K. M. (2011). Relaxation techniques for pain management in labour. *Cochrane Database of Systematic Reviews, 0*(12).

Conte, F., Terrin, G., Di Chiara, M., De Curtis, M., Aleandri, V., Scipione, A., . . . Bacchio, E. (2016). New architectural design of delivery room reduces morbidity in preterm neonates: a prospective cohort study. *BMC Pregnancy Childbirth, 16*, 63.

Crystle, C. D., Kegel, E. E., France, L. W., Brady, G. M., & Olds, R. E. (1980). The Leboyer method of delivery. An assessment of risk. *J Reprod Med, 25*(5), 267-271.

Davis, D., Hammond, A., Foureur, M., & Homer, C. S. E. (2013). Space, place and the midwife: Exploring the relationship between the birth environment, neurobiology and midwifery practice. *Women and Birth, 26*(4), 277-281.

Du, J. N., & Oliver, T. K., Jr. (1969). The baby in the delivery room. A suitable microenvironment. *Jama, 207*(8), 1502-1504.

Edwards, E. (2009). DISS A phenomenological analysis of women's choices, expectations and experiences when intending to give birth in a birth centre. *0*, 1.

Enkin, M., Rush, J., Burlock, S., Lambert, K., Loosley-Millman, M., & Hutchison, B. (1996). The effects of whirlpools baths in labor: a randomized, controlled trial. *Birth, 23*(3), 136-143.

Esposito, N. W. (1999). Marginalized women's comparisons of their hospital and freestanding birth center experiences: a contrast of inner-city birthing systems. *Health Care Women Int, 20*(2), 111-126.

Feldman, E., & Hurst, M. (1987). Outcomes and procedures in low risk birth: a comparison of hospital and birth center settings. *Birth, 14*(1), 18-24.

Fenwick, J., Foureur, M., Thomson, V., & Townsend, B. (2016). The birth bed: A qualitative study on the views of midwives regarding the use of the bed in the birth space. *Women Birth, 29*(1), 80-84.

Fischbacher, C., & Muthu, V. (2004). Free-standing midwife-led maternity units: a safe and effective alternative to hospital delivery for low-risk women? (Structured abstract). *Evidence-Based Healthcare and Public Health, 8*(2), 325-331.

Fleming, V., & Martin, C. H. (2011). The birth satisfaction scale. *Int J Health Care Qual Assur, 24*(2), 124-135.

Foureur, M., Homer, C. S. E., & Hammond, A. D. (2014). Messages from Space: An Exploration of the Relationship between Hospital Birth Environments and Midwifery Practice. *Herd-Health Environments Research & Design Journal, 7*(4), 81-95.

Gantar, I. Š., & Mole, H. (2011). Oxygen use in initial resuscitation of preterm infants. *Paediatria Croatica, Supplement, 55*, 189-194.

Gates, S., Hofmeyr, G. J., Sakala, C., & Hodnett, E. D. (2005). Continuous Support for Women During Childbirth. *Birth: Issues in Perinatal Care, 32*(1), 1-72.

Gibson, E., Kollath, C., & Leatherman, T. (2012). DISS Reinterpreting reproduction: An ethnography on discourses ideologies, and practices among midwifery participants in South Carolina. *3523121*, 302.

Gilbert, S., Newland, J., Rohlandt, D., & Haslam, A. (2012). The clocks of Malta: accuracy of clocks in the Women's Assessment Unit and Delivery Suite at Waikato Hospital. *N Z Med J, 125*(1366), 87-89.

Golden, S. S., & Sleutel, M. (1999). Fasting in labor: relic or requirement. *J Obstet Gynecol Neonatal Nurs, 28*(5), 507-512.

Gondry, J., Fontaine, C., Carpentier, E., Tourneux, P., Dubruque, E., Baumert, A., . . . Poupart, C. (2015). [Skin-to-skin care in the delivery room: impact of SpO2 monitoring]. *Arch Pediatr, 22*(2), 166-170.

Grunewald, C., Waldenstrom, U., & Gottvall, K. (2004). Safety of birth centre care: perinatal mortality over a 10-year period. *Bjog, 111*(1), 71-78.

Guinsburg, R., de Almeida, M. F. B., da Costa, J. O., Anchieta, L. M., & Freire, L. M. S. (2008). Material and human resources for neonatal resuscitation in public maternity hospitals in Brazilian state capitals. *Sao Paulo Medical Journal, 126*(3), 156-160.

Gülmezoglu, A. M., Hofmeyr, G. J., Nikodem, V. C., Nolte, A. G., & Wolman, W. (1998). Companionship by a lay labour supporter to modify the clinical birth environment: long-term effects on mother and child. *Curationis, 21*(1), 8-12.

Hamilton, N. A., Wallston, K. A., & Stevens, N. R. (2012). Perceived control and maternal satisfaction with childbirth: a measure development study. *J Psychosom Obstet Gynaecol, 33*(1), 15-24.

Hammond, A., Foureur, M., & Homer, C. S. E. (2014). The hardware and software implications of hospital birth room design: A midwifery perspective. *Midwifery, 30*(7), 825-830.

Hansmann, G. (2009). What the neonatologist would like to find in the delivery room *Neonatal Emergencies: A Practical Guide for Resuscitation, Transport and Critical Care of Newborn Infants* (Vol. 0, pp. 13-14).

Hazarika, J., & Das, B. K. (2015). Evaluation of health institution providing delivery services in seven districts of Assam. *Journal of Evolution of Medical and Dental Sciences-Jemds, 4*(15), 2513-2518.

Heath, B. J., Done, M., Balog, O., Ziccone, S., & Rosewarne, F. (1994). The effect of scavenging on nitrous oxide pollution in the delivery suite. *Aust N Z J Obstet Gynaecol, 34*(4), 484-486.

Hensley, M. J., Wlodarczyk, J. H., Brinsmead, M. W., & Rowley, M. J. (1995). Continuity of care by a midwife team versus routine care during pregnancy and birth: a randomised trial (Structured abstract). *Medical Journal of Australia, 163*(6), 289-293.

Hildingsson, I., Waldenstrom, U., & Rudman, A. (2006). Intrapartum and postpartum care in Sweden: women's opinions and risk factors for not being satisfied. *Acta Obstet Gynecol Scand, 85*(5), 551-560.

Hodnett, E. (2003). The effectiveness of continuous support during labour: impact of the hospital sub-culture. *International Nursing Perspectives, 3*(2), 95-97.

Hodnett, E. D. (2002). Pain and women's satisfaction with the experience of childbirth: a systematic review. *Am J Obstet Gynecol, 186*(5), S160-172.

Hodnett, E. D., Downe, S., & Walsh, D. (2012). Alternative versus conventional institutional settings for birth. *Cochrane Database of Systematic Reviews, 0*(8).

Hodnett, E. D., Gates, S., Sakala, C., & Hofmeyr, G. J. (2013). Continuous support for women during childbirth. *Cochrane Database of Systematic Reviews, 0*(7).

Hoffman, K., Rayburn, W., Goodlin, R., & Lorkovic, M. (1987). Alternative birth centers: a four year experience at the University of Nebraska Medical Center. *Nebr Med J, 72*(8), 286-288.

Hofmeyr, G. J., Chalmers, B., Nikodem, V. C., & Wolman, W. L. (1993). Postpartum depression and companionship in the clinical birth environment - a randomized, controlled-study. *American Journal of Obstetrics and Gynecology, 168*(5), 1388-1393.

Hofmeyr, G. J., Kramer, T., Nikodem, V. C., Wolman, W. L., & Chalmers, B. E. (1992). Companionship to modify the clinical birth environment: Effects on progress and perceptions of labour, and breastfeeding. *Obstetrical and Gynecological Survey, 47*(3), 162-164.

Holbert, D., Knobel, R., & Wimmer, J. (2005). Heat loss prevention for preterm infants in the delivery room. *Journal of perinatology : official journal of the California Perinatal Association, 25*(5), 304-308.

Holditch-Davis, D., & Knobel, R. (2007). Thermoregulation and heat loss prevention after birth and during neonatal intensive-care unit stabilization of extremely low-birthweight infants. *J Obstet Gynecol Neonatal Nurs, 36*(3), 280-287.

Holditch-Davis, D., & Knobel, R. (2010). Thermoregulation and heat loss prevention after birth and during neonatal intensive-care unit stabilization of extremely low-birthweight infants. *Adv Neonatal Care, 10*(5), S7-14.

Holland, C. V., Clark, R. B., Heien, J. B., & Strand, N. M. (1975). An improved infant resuscitation cart for the delivery room. *JOGN Nurs, 4*(4), 33-36.

Honda, M., Sakurai, H., Wada, M., Morita, K., Tamura, M., Kawasaki, H., . . . Kakei, H. (2016). Status of and problems concerning neonatal resuscitation in Japan in 2015. *Pediatr Int, 0*.

Hopkins, M., Kennedy, J., Jagger, C., MacVicar, J., Dobbie, G., & Owen-Johnstone, L. (1993). Simulated home delivery in hospital: a randomised controlled trial. *British journal of obstetrics and gynaecology, 100*(4), 316-323.

Hunter, L. P. (2009). A descriptive study of "being with woman" during labor and birth. *J Midwifery Womens Health, 54*(2), 111-118.

Hutton, E., McDonald, H., Murray-Davis, B., Rietsma, A., & Coubrough, M. (2014). Deciding on home or hospital birth: results of the Ontario Choice of Birthplace Survey. *Midwifery, 30*(7), 869-876.

Hyde, E. J., & Brighton, A. K. (2012). Accuracy and synchronisation of clocks between delivery suite and operating theatre. *N Z Med J, 125*(1366), 85-86.

Igarashi, T., Nakayama, T., Miyazaki, K., & Wakita, M. (2014). Birth environment facilitation by midwives assisting in non-hospital births: a qualitative interview study. *Midwifery, 30*(7), 877-884.

Iida, M., Horiuchi, S., & Porter, S. E. (2012). The relationship between women-centred care and women's birth experiences: a comparison between birth centres, clinics, and hospitals in Japan. *Midwifery, 28*(4), 398-405.

Jamas, M. T., Tanaka, A. A., & Hoga, L. K. (2011). Mothers' birth care experiences in a Brazilian birth centre. *Midwifery, 27*(5), 693-699.

James, L. S., & Dahm, L. S. (1972). Newborn temperature and calculated heat loss in the delivery room. *Pediatrics, 49*(4), 504-513.

Johnson, N., Gupta, J. K., & Johnson, V. A. (1991). Maternal positions during labor. *Obstet Gynecol Surv, 46*(7), 428-434.

Jones, S. (2005). Sensitive care for newborns. *International Journal of Childbirth Education, 20*(4), 8-13.

Joseph, R. (1990). Resuscitation at birth. *Singapore Med J, 31*(2), 166-170.

Kesti, T. J., & Ferraro, K. J. (2016). DISS Birthing against the mainstream: Women’s experiences and perceptions of out-of-hospital birth. *10131748*, 162.

Kiencke, P., Piroth, C., Gohring, U. J., Neuhaus, W., & Mallman, P. (2002). A psychosocial analysis of women planning birth outside hospital. *J Obstet Gynaecol, 22*(2), 143-149.

Kime, R., & Smales, O. R. (1978). Thermoregulation in babies immediately after birth. *Arch Dis Child, 53*(1), 58-61.

Koh, K. S., Peddle, L. J., Yung, S., & Greves, D. (1975). Experience with fetal monitoring in a university teaching hospital. *Can Med Assoc J, 112*(4), 455-456, 459-460.

Kramer, M., Gelfand, M., Klein, M. C., Papageorgiou, A., Westreich, R., Spector-Dunsky, L., & Elkins, V. (1983). [Satisfaction of couples towards birth when using a birthing room or the traditional delivery room.]. *Sante Ment Que, 8*(2), 47-54.

Kramer, T., Hofmeyr, G., Nikodem, V., Wolman, W., & Chalmers, B. (1991). Companionship to modify the clinical birth environment: effects on progress and perceptions of labour, and breastfeeding. *British journal of obstetrics and gynaecology, 98*(8), 756-764.

Laube, D. W. (1983). Experience with an alternative birth center in a university hospital. *J Reprod Med, 28*(6), 391-396.

Lee, A., Ulbricht, C., & Dorffner, G. (1998). Neural networks for recognizing patterns in cardiotocograms. *Artificial Intelligence in Medicine, 12*(3), 271-284.

Lee, A. H., Karkee, R., & Pokharel, P. K. (2014). Women's perception of quality of maternity services: a longitudinal survey in Nepal. *BMC Pregnancy Childbirth, 14*, 45.

Lee, H. C., & Lapcharoensap, W. (2016). Temperature management in the delivery room and during neonatal resuscitation. *NeoReviews, 17*(8), e454-e462.

Lehrman, E. (1988). DISS A theoretical framework for nurse-midwifery practice. *0*, 195 p-195 p.

Leone, T. A., Finer, N. N., Halamek, L. P., & Rich, W. (2012). The delivery room of the future: the fetal and neonatal resuscitation and transition suite. *Clin Perinatol, 39*(4), 931-939.

Liu, B., Seed, P., O'Sullivan, G., Hart, D., & Shennan, A. (2009). Effect of food intake during labour on obstetric outcome: randomised controlled trial. *BMJ (Clinical research ed.), 338*, b784.

Lubic, R. W. (1988). The freestanding birth center as a model for alternative maternity care. *NLN Publ, 0*(15), 53-66.

McCourt, C., Sandall, J., Rance, S., & Rayment, J. (2014). Health Services and Delivery Research. *An ethnographic organisational study of alongside midwifery units: a follow-on study from the Birthplace in England programme, 0*.

McNelis, M. (2013). Women's experiences of care during labour in a midwifery-led unit in the Republic of Ireland. *British Journal of Midwifery, 21*(9), 622-631.

Mistrangelo, E., Morano, S., Costantini, S., Pastorino, D., Ragni, N., Cerutti, F., & Benussi, M. (2007). Outcomes of the first midwife-led birth centre in Italy: 5 years' experience. *Arch Gynecol Obstet, 276*(4), 333-337.

Nelson, E. J., Parrott, R. L., & Condit, C. M. (1996). The American experience of childbirth: Toward a range of safe choices *Evaluating women's health messages: A resource book* (Vol. 0, pp. 109-123, Chapter xiv, 445 Pages).

Newburn, M. (2012). The best of both worlds--parents' motivations for using an alongside birth centre from an ethnographic study. *Midwifery, 28*(1), 61-66.

Noori, N. M., Teimouri, A., Boryri, T., & Yaghobinia, F. (2016). The perception of primiparous mothers of comfortable resources in labor pain (a qualitative study). *Iran J Nurs Midwifery Res, 21*(3), 239-246.

O'Donnell, C. P., McCarthy, L. K., & Hensey, C. C. (2012). In vitro effect of exothermic mattresses on temperature in the delivery room. *Resuscitation, 83*(10), e201-202.

Oliveira, S., Nobre, M., & Silva, F. (2009). A randomised controlled trial evaluating the effect of immersion bath on labour pain. *Midwifery, 25*(3), 286-294.

Osler, M., Holstein, B. E., Kamper-Jorgensen, F., & Poulsen, E. F. (1979). [Birth environment investigation 1976--1977. I. Material, methods, pregnancy and antenatal preparation]. *Ugeskr Laeger, 141*(26), 1779-1783.

Osler, M., Holstein, B. E., Kamper-Jorgensen, F., & Poulsen, E. F. (1979). [Birth environment investigation 1976--1977. II. Normal deliveries]. *Ugeskr Laeger, 141*(26), 1783-1786.

Osler, M., Holstein, B. E., Kamper-Jorgensen, F., & Poulsen, E. F. (1979). [Birth environment investigation. III. Puerperium, infants in neonatal departments and informative material]. *Ugeskr Laeger, 141*(26), 1786-1791.

Palmer, S., Abram, S. E., Maitra, A. M., & Jahn, E. P. (1984). Obstetric side brace for the delivery room table. *Anesth Analg, 63*(11), 1046-1047.

Papadopoulos, I., Martensson, L. B., Hadjigeorgiou, E., Kouta, C., & Papastavrou, E. (2012). Women's perceptions of their right to choose the place of childbirth: an integrative review. *Midwifery, 28*(3), 380-390.

Paul, J., Carr, V., Symon, A. G., Dugard, P., & Butchart, M. (2011). Care and environment in midwife-led and obstetric-led units: a comparison of mothers' and birth partners' perceptions. *Midwifery, 27*(6), 880-886.

Paul, N., Girish, M., Pandey, P., Mujawar, N., Gotmare, P., & Punia, S. (2013). Impact and feasibility of breast crawl in a tertiary care hospital. *Journal of Perinatology, 33*(4), 288-291.

Payne, D., Wilson, S., Smythe, E., Gunn, J., Hunter, M., Crowther, S., & Couper, J. M. (2016). Midwifing the notion of a 'good' birth: a philosophical analysis. *Midwifery, 37*, 25-31.

Petravage, J. B. (1983). Outcomes of three birthing rooms. *J Fam Pract, 16*(5), 929-933.

Pinjaroen, S., Tiansawad, S., Chunuan, S., Somsap, Y., Nangham, S., & Jeamamornrat, A. (2007). An evaluation of childbirth policy in Thailand: a case study in the southern part of Thailand (part 1). *Thai Journal of Nursing Research, 11*(4), 227-238.

Posey, A. D. (2000). *Perceived maternal competence of postpartum women giving birth in an alternative setting.* (1400041).

Ratcliffe, J., Boulton, M., & Longworth, L. (2001). Investigating women's preferences for intrapartum care: home versus hospital births. *Health Soc Care Community, 9*(6), 404-413.

Rimkoute, A., & South, T. (2013). Why VBAC birthplace matters: A literature review. *British Journal of Midwifery, 21*(5), 364-370.

Rogers, C., Harman, J., & Selo-Ojeme, D. (2011). Perceptions of birth in a stand-alone centre compared to other options. *British Journal of Midwifery, 19*(4), 237-244.

Ruble, D. N., Flett, G. L., Fleming, A. S., & Anderson, V. (1988). Place of childbirth influences feelings of satisfaction and control in first-time mothers. *Journal of Psychosomatic Obstetrics & Gynecology, 8*(1), 1-17.

Russo, A., Perlman, J., Huynh, T., Torres, L., Grunebaum, A., Perlmutter, D., . . . Handrinos, S. (2014). Reducing Hypothermia in Preterm Infants Following Delivery. *Pediatrics, 133*(4), E1055-E1062.

Ryan, M., Hundley, V., & Graham, W. (2001). Assessing women's preferences for intrapartum care. *Birth: Issues in Perinatal Care, 28*(4), 254-263.

Sacks, S. R., & Donnenfeld, P. B. (1984). Parental choice of alternative birth environments and attitudes toward child-rearing philosophy. *Journal of Marriage and the Family, 46*(2), 469-475.

Schachter, M., Cannoodt, L., & Sieverts, S. (1982). Alternatives to the conventional in-hospital delivery: the Childbearing Center experience. *Acta Hosp, 22*(4), 324-339.

Schmied, V., Dahlen, H., & Priddis, H. (2012). What are the facilitators, inhibitors, and implications of birth positioning? A review of the literature. *Women and Birth, 25*(3), 100-106.

Schulling, S. K. (1988). *Choice of birth site as it relates to locus of control.* (1334517).

Schwartz, L. (1974). *The environment of birth: An exploration of the process of pregnancy, labor and delivery.* (0).

Scrymgeour, C., & Myers, S. J. (2012). DISS Determinants of Women's Choice of Birth Center Versus Home or Hospital Birth in Alberta, Canada - January 2010 - June 2011. *1514961*, 70.

Sedin, G. (2012). Physical environment: The thermal environment. *Neonatology: A Practical Approach to Neonatal Diseases, 0*, 178-188.

Singh, P. K., Chauhan, S., Prasad, P. L., & Gahalaut, P. (2013). Correlation of pulse oximetry and apgar scoring in the normal newborns. *J Clin Neonatol, 2*(1), 20-24.

Singh, P. P., Dimich, I., Jhaveri, M., Adell, A., Hendler, M., & Sonnenklar, N. (1991). Evaluation of oxygen saturation monitoring by pulse oximetry in neonates in the delivery system. *Can J Anaesth, 38*(8), 985-988.

Skibsted, L., & Lange, A. P. (1990). Breast-feeding in a Danish alternative birth center compared with the obstetrical ward. *Early Hum Dev, 21*(2), 115-124.

Stewart, P., Hillan, E., & Calder, A. A. (1983). A randomised trial to evaluate the use of a birth chair for delivery. *Lancet, 1*(8337), 1296-1298.

Stramrood, C. A. I., Weijmar Schultz, W. C. M., van Pampus, M. G., Vingerhoets, A. J. J. M., Paarlberg, K. M., Huis In 't Veld, E. M. J., & Berger, L. W. A. R. (2011). Posttraumatic stress following childbirth in homelike- and hospital settings. *Journal of Psychosomatic Obstetrics & Gynecology, 32*(2), 88-97.

Sychareun, V., Hansana, V., Phengsavanh, A., Somphet, V., Xayavong, S., & Popenoe, R. (2012). Reasons rural Laotians choose home deliveries over delivery at health facilities: a qualitative study. *BMC Pregnancy Childbirth, 12*, 86.

Tabatabaei, S. M., & Moudi, Z. (2016). Birth outcomes in a tertiary teaching hospitals and local outposts: a novel approach to service delivery from Iran. *Public Health, 135*, 114-121.

Tabatabaei, S. M., Vedadhir, A., Moudi, Z., & Tabatabaie, M. G. (2014). Safe Delivery Posts: An intervention to provide equitable childbirth care services to vulnerable groups in Zahedan, Iran. *Midwifery, 30*(10), 1073-1081.

Takács, L., Seidlerová, J. M., Šulová, L., & Hoskovcová, S. H. (2015). Social psychological predictors of satisfaction with intrapartum and postpartum care-what matters to women in Czech maternity hospitals? *Open Medicine, 10*(1), 119-127.

Thompson, L. M., & Beam, N. K. (2016). Women and men's preferences for delivery services in rural Ethiopia. *10133409*, 193.

Tjalma, W. A., & Eelen, C. M. (2002). The development of subcutaneous emphysema after delivery on a specially designed chair. *Clin Exp Obstet Gynecol, 29*(1), 22.

Townsend Fullerton, J. D. (1982). The choice of in-hospital or alternative birth environment as related to the concept of control. *Journal of Nurse-Midwifery, 27*(2), 17-22.

Tracy, M., Monk, A., Foureur, M., Grigg, C., & Tracy, S. (2014). Evaluating Midwifery Units (EMU): a prospective cohort study of freestanding midwifery units in New South Wales, Australia. *BMJ Open, 4*(10), e006252.

Tranmer, J., Gyte, G. M., & Singata, M. (2013). Restricting oral fluid and food intake during labour. *Cochrane Database of Systematic Reviews, 0*(8).

Turner, L. R., Macfarlane, A. J., & Rocca-Ihenacho, L. (2014). Survey of women's experiences of care in a new freestanding midwifery unit in an inner city area of London, England: 2. Specific aspects of care. *Midwifery, 30*(9), 1009-1020.

Turner, L. R., Roth, C., Macfarlane, A. J., & Rocca-Ihenacho, L. (2014). Survey of women's experiences of care in a new freestanding midwifery unit in an inner city area of London, England. 1: Methods and women's overall ratings of care. *Midwifery, 30*(9), 998-1008.

Turner, M., Glazener, C. M., Lang, G. D., Mollison, J., Hundley, V. A., Cruickshank, F. M., . . . Blyth, D. (1995). Satisfaction and continuity of care: staff views of care in a midwife-managed delivery unit. *Midwifery, 11*(4), 163-173.

Waldenstrom, U., & Gottvall, K. (1991). A randomized trial of birthing stool or conventional semirecumbent position for second-stage labor. *Birth, 18*(1), 5-10.

Waldenstrom, U., & Nilsson, C. A. (1994). Experience of childbirth in birth center care. A randomized controlled study. *Acta Obstet Gynecol Scand, 73*(7), 547-554.

Waldenström, U. (1998). Continuity of carer and satisfaction. *Midwifery, 14*(4), 207-213.

Waldenström, U., El-Khouri, B., & Rudman, A. (2007). Women's satisfaction with intrapartum care--A pattern approach. *Journal of Advanced Nursing, 59*(5), 474-487.

Walsh, D. (2004). Birth centre care: a review of the literature. *Birth Issues, 13*(4), 129-134.

Walsh, D. J. (2006). 'Nesting' and 'Matrescence' as distinctive features of a free-standing birth centre in the UK. *Midwifery, 22*(3), 228-239.

Walton, C., Yiannousiz, K., & Gatsby, H. (2005). Promoting midwifery-led care within an obstetric-led unit. *British Journal of Midwifery, 13*(12), 750-755.

Wang, Y. K., Chen, K. H., & Chen, L. R. (2014). Contamination of medical charts: an important source of potential infection in hospitals. *PLoS One, 9*(2), e78512.

Vedam, S. (2003). Home birth versus hospital birth: questioning the quality of the evidence on safety. *Birth, 30*(1), 57-63.

Vellone, E., Alvaro, R., Amato, E., & D'Aliesio, L. (2009). The positive effects of father's attendance to labour and delivery: a quasi experimental study. *International Nursing Perspectives, 9*(1), 5-10.

Westerink, J. M. (1986). Inter-relationships between environmental factors, personal experiences and behaviour following childbirth: A longitudinal study. *0*, 1-394.

Vidyasagar, D., Patil, V. D., & Bhandankar, M. (2012). Differences in thermal adaptation of infants delivered at primary or tertiary care facilities in India. *J Perinatol, 32*(7), 502-507.

Vidyasagar, D., Patil, V. D., & Bhandankar, M. (2012). Postnatal thermal adaptation and the effects of seasonal variations on temperature trends leading to cold stress in newborn infants in rural India. *Journal of Neonatal-Perinatal Medicine, 5*(4), 373-379.

Wiegers, T. A., & Borquez, H. A. (2006). A comparison of labour and birth experiences of women delivering in a birthing centre and at home in the Netherlands. *Midwifery, 22*(4), 339-347.

Worth, J. (1999). Neonatal sensitisation to latex. *Positive Health, 0*(41), 27-32.

Worth, J. (2000). Neonatal sensitization to latex. *Med Hypotheses, 54*(5), 729-733.

Wu, C. J., & Chung, U. L. (2003). The decision-making experience of mothers selecting waterbirth. *J Nurs Res, 11*(4), 261-268.

Yagoub, S. O., & El Agbash, A. (2010). Isolation of Potential Pathogenic Bacteria from the Air of Hospital-Delivery and Nursing Rooms. *Journal of Applied Sciences, 10*(11), 1011-1014.
